# Acute Demyelinating Encephalomyelitis Post-COVID-19 Vaccination: A Case Report and Literature Review

**DOI:** 10.3390/diseases10010013

**Published:** 2022-02-20

**Authors:** Khalid Al-Quliti, Ahmad Qureshi, Mohammed Quadri, Babar Abdulhameed, Alhanouf Alanazi, Rakan Alhujeily

**Affiliations:** 1 Neurology Division, Department of Medicine, College of Medicine, Taibah University, Al-madinah Almunwwarah 42353, Saudi Arabia; 2 Intensive Care Unit, National Guard Hospital, Al-madinah Almunwwarah 42324, Saudi Arabia; qureshiah@ngha.med.sa (A.Q.); hameedquadri@yahoo.com (M.Q.); drbabar20@gmail.com (B.A.); 3 College of Medicine, Taibah University, Al-madinah Almunwwarah 42353, Saudi Arabia; alhanouf.nzy@gmail.com (A.A.); rakanma97@gmail.com (R.A.)

**Keywords:** acute demyelinating encephalomyelitis, COVID-19, vaccination

## Abstract

New advancements in the medical community have rapidly occurred with the development of medical information across the globe during the COVID-19 pandemic. Several vaccine manufacturers were able to obtain clearance to administer vaccines in selected age groups and for those at high risk for COVID-19 complications. As vaccines became more readily available, there was a significant effort supported by scientific information to get people vaccinated to boost herd immunity. Acute demyelinating encephalomyelitis (ADEM) is a rare autoimmune disease, causing demyelination in the brain and spinal cord, presenting as monophasic, acute-onset, and rapidly progressive multifocal neurological deficits. A wide variety of precipitating factors can trigger ADEM, and it has long been known to be a rare adverse event following some types of vaccinations including rabies, diphtheria–tetanus–polio, smallpox, measles, mumps, rubella, pertussis, influenza, and hepatitis B vaccines. Recently, ADEM has also been associated with COVID-19 infection and (very rarely) with COVID-19 vaccination. We have a 56-year-old female who was not known to have any medical issues. She voluntarily received her first COVID-19 vaccination (AstraZeneca) ten days after immunization; she developed weakness of the lower limbs and slurred speech. She tested negative for COVID-19, and a brain MRI showed T2-weighted white-matter hyperintense lesions suggesting acute demyelinating encephalomyelitis. She was managed with pulse-dose steroids, which resulted in a marked improvement in her symptoms, and discharged in a stable condition. Physicians should be aware of this neurological disorder and the management options for better patient care and outcomes.

## 1. Introduction

Acute demyelinating encephalomyelitis (ADEM) is an intense inflammatory neurological disorder that occurs with many etiologies. It is commonly a monophasic disorder with acute-onset and progressive multifocal neurological deficits. Its exact incidence is unknown. Population-based studies estimate the prevalence at 3.3 per 100,000 people. It is more common in children and young adults under 20 years of age and affects men more than women (ratio, 1.3:1) These disorders typically occur 1–2 weeks post-infection or post-vaccination, but the presentation can be delayed by up to 3 months [1]. Case definitions for ADEM have been established by the Brighton Collaboration [2], characterized by clinical and radiological abnormalities that are best identified by magnetic resonance imaging (MRI) of the brain, brainstem, and spinal cord. The MRI results for the T2-weighted images, fluid-attenuated inversion recovery (FLAIR) sequence, and Gadolinium contrast-enhanced images are the most useful. The MRI results consist of lesions in the deep white matter and subcortical area of variable sizes and locations, with similarity on both sides of the brain, brainstem, and spinal cord. Most cases of ADEM are post-viral or bacterial infection or, less often, post-vaccination. A number of vaccines have been implicated as causative agents such as influenza, HPV, PCV, rabies, diphtheria–tetanus–pertussis, polio, smallpox, measles, mumps, rubella, Japanese B encephalitis, influenza, and hepatitis B [1,3]. Pellegrino et al. [3], based on their review of the Vaccine Adverse Event Reporting System (VAERS) and EudraVigilance post-authorization module (EVPM), reported that HPV (1 and 2) and the flu mostly accompanied ADEM, and they were considered together for about a third of cases. ADEM has also been reported with COVID-19 infection, and there are a few reports of suspected cases with COVID-19 vaccination. We report a suspected case of ADEM that may have a relation to COVID-19 vaccination, with a good response to IV-steroid therapy. Approval was obtained from the relevant regulatory committee (41-H01-2021), and informed consent was obtained.

## 2. Case Presentation

**Figure 1:**
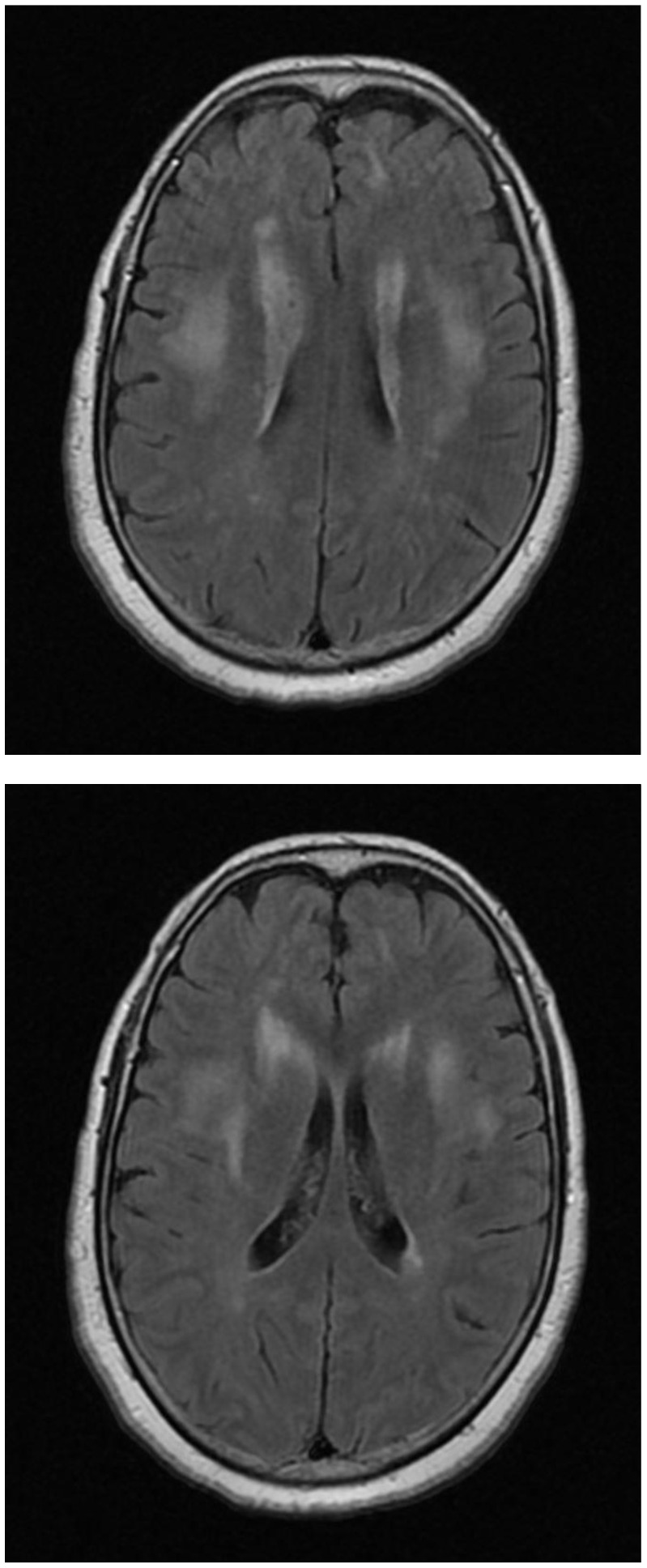
MRI of brain: fluid-attenuated inversion recovery (FLAIR) sequences, showing multifocal, bilateral, asymmetric, multiple hyperintense lesions in the deep and subcortical white matter. The thalami and basal ganglia.

A 56-year-old female who was not known to have any medical illness, with no history of upper-respiratory-tract symptoms, was vaccinated for COVID-19 (AstraZeneca); 10 days later, she presented to the emergency department (ED) with gradual discomfort followed by generalized weakness mainly affecting the lower limbs and proximal and distal muscles, with lower-extremity myalgias. Her clinical exam as well as lab work was normal; therefore, the patient was discharged. A COVID-19 PCR was also negative. She was prescribed a proton-pump inhibitor (omeprazole, 40 mg, orally) and acetaminophen PRN to relieve her aches and pain. The patient then developed worsening lower-limb weakness, associated with difficultly in the articulation of speech; she needed assistance to ambulate, and she had anorexia during this time. She did not have any history of polydipsia, and she had no loss of sensation or urinary/fecal incontinence. She again presented to the ED with profound weakness of the lower extremities and some dysmetria on the left side. She was fully alert, awake, and oriented. The patient, vitally, had high blood pressure, at 190/95 mm Hg, a pulse of 95 beats per minute, a temperature of 37 °C, and an oxygen saturation of 94% on room air. Subsequently, her BP improved to 156/74 mm Hg spontaneously. The patient’s BMI was 28.3 kg/m^2^. On physical examination, her cardiac, respiratory, and abdominal examinations were normal. On neurological examination, she was fully conscious; alert; and oriented to time, place, and person. She had a normal Glasgow Coma Scale score, at 15/15. Her pupils were normal in size and reactive to light. There was a bilateral-adduction-gaze deficit, with a normal gag reflex. There were signs of meningism, with stiffness of the neck. Her motor exam showed weakness with a power of 4/5 in both upper limbs and 3/5 weakness in both lower limbs. There were diminished reflexes without a loss of sensation. The gait was difficult to examine. The laboratory work-up was remarkable, with a serum sodium of 116 mEq/L, urinary sodium level of 64, serum osmolality of 236, and urine osmolality of 700, consistent with SIADH. The thyroid profile and cortisol levels were within the normal ranges. Lumbar puncture showed clear cerebrospinal fluid, with CSF protein elevated, at 1.76, and CSF glucose elevated, at 4.62; the CSF WBC count was 1 and the RBC count was 7; the CSF differential cells were CSF segs, 20%; CSF mono, 64%; and lymphocytes, 16%. The urinary electrolytes showed a sodium level of 64, Cl level of 89, and K level of 36. The chest X-ray was unremarkable. MRI images: the T2 and FLAIR sequences demonstrated large multifocal, bilateral, asymmetric, multiple hyperintensities in the subcortical and deep white matter involving the basal ganglia with no contrast enhancement, as shown in Figure 1.

She was managed with hypertonic saline at 2% and very slow sodium correction over the next 24 h at a rate < 0.5 mEq/h and eventually improved to 133 mEq/L over 1 week. Omeprazole was discontinued. She received steroids (pulse dose) using 1 g of methylprednisolone for 5 days, accompanied by physical and occupational therapy. This led to the complete resolution of her symptoms; the neck stiffness and bilateral-adduction-gaze deficit were resolved, as well as minimal improvement in her lower limbs’ weakness being observed. During the hospital stay, she continued to improve and was able to mobilize freely without assistance, and she was discharged from hospital with a plan for a follow-up brain MRI after six months.

## 3. Discussion

Although adenovirus has been associated with acute necrotizing encephalitis, there have been no case reports linking adenovirus vectors to ADEM to the best of our knowledge [4]. This case report focuses on the importance of the neurological workup of patients post-vaccination who present with unexplained neurological findings. A follow-up MRI may confirm ADEM, as the lesions may improve or remain stable over time. However, if new lesions are identified on follow-up MRI scans, then other differential diagnoses such as multiple sclerosis can be considered. However, in this patient, the symptoms started early, while, generally, in the case of ADEM, the symptoms start from 1–2 weeks up to 3 months after infection or vaccination. Thus, other differential diagnoses with different pathology such as hypoxia and metabolic and toxic cases were considered as well. Once the diagnosis is established, patients whose symptoms are mild can be watched, while those with significant or persistent symptoms can be provided with intravenous steroids followed by a prednisolone taper over 4–6 weeks. Those who fail to respond to pulse-dose steroids can be managed by IVIG for 5 days, while plasmapheresis is reserved for patients who do not respond to both steroids and IVIG [5,6,7]. While the overall mortality from ADEM is about 5%, a great majority of patients (70–90%) show significant improvements in neurological deficits over a period of 1–6 months [8].

In addition, other treatment modalities include intravenous cyclophosphamide and mitoxantrone, which can be tried if the above treatment modalities fail. Once an ADEM diagnosis is determined, further immunization can be avoided or delayed for 6 months, with a follow-up brain MRI. It is best to avoid immunization for at least 6 months, as it can cause a relapse to multiphasic disseminated encephalomyelitis (MDEM), which is defined as an acute demyelinating CNS disease, which can occur within 4 weeks of the onset of the original symptoms [1,9].

ADEM lesions can be hemorrhagic, as in the case of a 53-year-old male admitted for respiratory failure due to COVID-19 who then developed ADEM. According to FLAIR, there was little hemorrhage intraventricularly, along with occipital horns and parenchymal small hemorrhages located superficially in the parietal gyri, right and left superior frontal lobes, and right occipital lobe [10].

ADEM lesions can be associated with hemorrhagic pathology in the case of severe infection with respiratory failure due to COVID-19. A hemorrhage can extend intraventricularly along the occipital horns, frontal lobes, and occipital lobe [10]. It is important to know that poor prognostic factors are advanced age, female gender, the functional impairment degree at clinical onset, spinal cord involvement, the level of CSF protein, a poor response to corticosteroids, and peripheral-nerve-system damage. Functional impairment can be evaluated using the Scripps neurological rating scale (SNRS), which can examine mood, mentation, visual acuity, eye movement, lower cranial nerves, sensory/motor functions, reflexes, Babinski sign, gait, cerebellar function, and bladder/bowel functions. The SNRS score ranges from 10 to 100; when the score is high, it indicates a better function [1]. ADEM has been reported in a child post-COVID-19 infection and, for that reason, should be considered a treatable cause of multifocal neurological deficits [11].

## 4. Literature Review

Acute disseminating encephalomyelitis is defined as a demyelinating disease of the CNS that presents as a monophasic disorder with encephalopathy and neurological symptoms that are considered multifocal [12]. The incidence of ADEM in the United Kingdom is 0.23/100,000 [10]. Demyelination areas can be found by histopathology, or the presence of one or more multifocal findings related to the CNS (encephalopathy, focal cortical signs, cranial-nerve abnormalities, visual-field defects, positive primitive reflexes, motor weakness, sensory abnormalities, deep tendon reflex alterations, or cerebellar dysfunction), MRI findings (multifocal lesions in the white matter on T2-weighted, diffusion-weighted imaging, or FLAIR), and monophasic pattern of illness (the absence of relapse within 3 months of the symptomatic nadir) [1].

### 4.1. Etiology and Clinical Features

The disease can mostly be triggered by several viral infections or post-infectious causes, such as the hepatitis A, B, or C virus; measles; mumps; rubella; VZV; CMV; EBV; HSV; influenza A or B; coxsackie virus; HIV; HHV-6; human T-cell lymphotropic virus-1 (HTLV-1); Rocky Mountain spotted fever; vaccinia; and human coronaviruses. It can also be caused by bacterial infections; the most common is mycoplasma pneumoniae. Other pathogens involved are chlamydia, borrelia, leptospira, legionella, campylobacter, and group A beta-hemolytic streptococci [1,13].

Another factor involved in causing ADEM is post-vaccination complications, such as those from influenza, HPV, PCV, rabies, diphtheria–tetanus–polio, smallpox, measles, mumps, rubella, Japanese B encephalitis, pertussis, influenza, and hepatitis B vaccines [1].

In a recently reported case based on antemortem medical records and the results of a postmortem investigation, the cause of death was registered as “ADEM in the setting of recent Astra Zeneca COVID-19 vaccination” [13]. ADEM can present in many ways including focal/multifocal neurological deficits, optic neuritis, seizures, and an altered mental status [14].

### 4.2. Neuroimaging and Histopathology

The typical hallmark for ADEM in MRI is asymmetrically bilateral brain lesions in either the supratentorial or infratentorial white matter that present as a hyperintense lesion in T2-weighted and FLAIR sequences [10]. Ghali et al. demonstrated that a biopsy enables a definitive diagnosis of ADEM [14]. The lesions can be bilateral and asymmetrical, involving the supratentorial cerebral white matter, posterior fossa (brainstem and cerebellum), or spinal cord [14].

### 4.3. Management Options and Prognosis

Patients diagnosed with ADEM who are mildly symptomatic should be observed. Patients with severe and persistent symptoms can be managed with IV steroids followed by a prednisolone taper over 4–6 weeks [15,16,17]. For those who fail to respond to the IV steroids, IVIG for 5 days can be considered. Plasmapheresis is reserved for those patients who do not respond to both steroids and IVIG [7]. A spontaneous improvement to full recovery can take up to 1–6 months in about 50 to 75% of cases [1].

## 5. Conclusions

Acute demyelinating encephalomyelitis (ADEM) is a neurological disorder considered to be inflammatory in nature, with several etiologies, that typically occurs 1–2 weeks after bacterial or viral infections, or vaccination. Several vaccines have been implicated as causative agents. We report this case of suspected ADEM that may have a relation to a COVID-19 vaccination that employs adenovirus as a vector.

## Data Availability

Not applicable.
